# Patterns of symptom deterioration can support multimorbidity management in COPD: Perspectives of patients and healthcare professionals

**DOI:** 10.1371/journal.pone.0338888

**Published:** 2025-12-17

**Authors:** Sanne H. B. van Dijk, Marjolein G. J. Brusse-Keizer, Bente Rodenburg, Anke Lenferink

**Affiliations:** 1 Health Technology & Services Research, Technical Medical Center, University of Twente, Enschede, The Netherlands; 2 Department of Pulmonary Medicine, Medisch Spectrum Twente, Enschede, The Netherlands; 3 Medical School Twente, Medisch Spectrum Twente, Enschede, The Netherlands; All India Institute of Medical Sciences, INDIA

## Abstract

**Introduction:**

Comorbidities significantly complicate COPD management. Remote monitoring could aid real-time disease and symptom management, assisting both patients with multimorbidity and healthcare professionals (HCPs). This study aimed to explore how insight in patterns of symptom deterioration, derived from remote monitoring, could enhance multimorbid COPD management as perceived by patients and HCPs.

**Methods:**

Using daily symptom data collected via a mobile diary in the prospective RE-SAMPLE cohort study, patterns of symptom deterioration of COPD, chronic heart failure, anxiety, and depression were visualized per patient (follow-up duration of ≥4 months). Semi-structured individual interviews were conducted with Dutch patients with COPD and ≥1 comorbidity, and with HCPs from pulmonology, cardiology, and medical psychology who were involved in care for patients with multimorbidity. Interviews addressed current multimorbid COPD management, its challenges, and the way pattern visualizations of symptoms deterioration could support disease management. Transcripts were thematically analyzed using an inductive approach.

**Results:**

7 patients (69–80 years, 4 men) and 7 HCPs were interviewed in the hospital (patients and HCPs), at home (patients) or online (HCPs). Three overarching themes were identified, representing the elements of multimorbid COPD management that could be supported by the pattern visualizations: 1) relationship between diseases, 2) decision-making, and 3) self-management. According to patients and HCPs, pattern visualizations can be an informative source to explain the relation between COPD and comorbidities, function as a conversation starter facilitating communication between patients and HCPs as well as between medical disciplines, and educate patients in adequately recognizing their care needs.

**Conclusion:**

Three elements of personalized multimorbid COPD management were identified through qualitative analysis, which can all be supported by visualizing patterns of symptom deterioration via remote monitoring. The visualizations could enhance patients’ understanding of their diseases, improve shared decision-making, improve in-hospital multidisciplinary collaboration, and support multimorbid COPD (self-)management.

## Introduction

Chronic obstructive pulmonary disease (COPD) is a progressive lung disease which affects approximately 10% of the global population and accounts for over 3 million deaths annually [1]. COPD is responsible for considerable decline in quality of life of patients [2], mainly due to its symptoms such as dyspnea, coughing, and fatigue [3]. The average patient with COPD also suffers from multiple chronic comorbidities [4], including cardiovascular, metabolic, orthopedic, and mental diseases [5]. COPD is characterized by episodes of sudden major increases of symptoms requiring additional medical treatment, called acute exacerbations of COPD (AECOPD) [3]. In presence of certain comorbidities, it is challenging to accurately differentiate AECOPDs from comorbid flare-ups, such as acute heart failure or a panic attack due to overlapping symptoms [6,7]. Additionally, AECOPDs regularly occur simultaneously with comorbid flare-ups [8], making it even more complex to offer adequate care in such cases.

Although COPD guidelines acknowledge that considering multimorbidity (i.e., co-occurrence of ≥2 chronic diseases in one individual [9]) is essential in treatment [3], in practice, diseases are often targeted separately [10]. Although healthcare professionals (HCPs) express the need to address multimorbidity more, they report a lack of support due to fragmented (i.e., discipline-specific) clinical guidelines [11,12]. This may lead to contradictory treatment assigned by different disciplines [10], unwanted side-effects or interactions with other diseases [13], and an increased treatment burden (including polypharmacy) for the patient resulting in adherence problems [13–15]. Also patients with COPD themselves struggle with multimorbidity: they find it difficult to differentiate acute deterioration of symptoms from normal fluctuation of symptoms or from other diseases, delaying seeking appropriate care [16] or initiating self-treatment timely [17]. Delay of appropriate (self-)treatment is proven to lead to a prolonged recovery time [18,19], potentially even requiring hospitalization [18].

To overcome such problems regarding disease management in patients with multimorbid COPD, it is essential to tailor care to the individual patient [10[, given that various combinations of chronic diseases lead to diverse care needs among these patients [20,21]. Not surprisingly, it can be hard for HCPs and patients themselves to adequately recognize one’s exact care need and define a personalized disease management strategy. Remote monitoring of multiple diseases simultaneously could possibly aid this process [22], as it allows for real-time measurement of symptoms and disease deterioration of COPD as well as its comorbidities. Visualization techniques can play a key role by helping to identify patient-specific trends and symptom deterioration patterns over time [8,23]. Both HCPs and patients may gain valuable insights into deterioration of and relations between COPD and comorbidities through remote monitoring via such pattern visualizations, possibly leading to timely recognition of care needs and (seeking) earlier and appropriate treatment.

However, there is still little knowledge regarding how insights into patterns of disease deterioration through remote monitoring could contribute to improvements in multimorbid disease management [8]. The aim of this study was, therefore, to explore how visualizations of patterns of COPD and comorbid symptom deterioration could support personalized care in multimorbid COPD management, as perceived by patients with multimorbidity and HCPs across various medical disciplines.

## Methods

This study was designed as a mixed-methods study, since it was assumed that qualitative methodology used to answer the research question would be best supported by visualizations originating from the quantitative data. It is therefore considered a so-called “qualitative dominant mixed-methods” study, from the view that the research aim can best be met through a qualitative approach (i.e., interviews) while recognizing that the addition of quantitative data (i.e., pattern visualizations) will benefit the findings [24]. The methods section will describe the methodology in chronological order including both the quantitative and qualitative parts, respectively. R version 4.3.1 [25] was used for the visualizations used in this study. All important aspects of the current study were reported following the consolidated criteria for reporting qualitative research (COREQ) checklist [26] (see S1 File).

### Study design & participants

This study was conducted in a teaching hospital in Twente, the Netherlands as part of an international prospective cohort study (the RE-SAMPLE study [27], see Text box 1). The RE-SAMPLE study was approved by Medical Ethical Committee Twente (NL77763.100.21). The current interview study’s protocol was approved by the hospital (K21-20) and the University of Twente Humanities & Social Sciences Ethics Committee (241013). All participants provided written informed consent before participating in this study. By means of semi-structured in-depth individual interviews with patients as well as HCPs, perspectives on multidisciplinary care for patients with COPD and comorbidities were collected. Dutch patients participating in RE-SAMPLE between January 2022 and November 2024 were recruited for the current study purposively between 4 November 2024 and 20 December 2024 based on I) the reported deterioration of symptoms of chronic heart failure (CHF), anxiety, and/or depression (i.e., ≥ 1 AECOPD or comorbid flare-up & increased symptoms of COPD as well as ≥1 comorbidity), and II) a minimal follow-up period of four months. HCPs were recruited from various relevant medical disciplines (i.e., pulmonology, cardiology, medical psychology) and professions (i.e., physician, specialized nurse, psychologist) and needed to be involved in the RE-SAMPLE study and/or care for patients with multimorbidity. All participants signed informed consent before participation in the interviews.

### Visualizing patterns of symptom deterioration

Symptoms of COPD, CHF, anxiety, and depression were daily collected until patient dropout or otherwise until November 2024 via individualized e-diaries (Text box 1). The e-diary data were used to visualize patterns (i.e., timelines) of symptom deterioration. Missing e-diary data of up to three consecutive observations (i.e., days) were imputed using a combination of the last observation carried forward and the next observation carried backward, similarly to an earlier study [28].Given the relatively narrow (i.e., daily) time intervals between the e-diary data points, consecutive values are generally highly correlated. Furthermore, symptom variability is better preserved this way compared with other methods such a mean imputation [29]. Hence, this imputation method was considered appropriate. Additionally, AECOPDs (e.g., extra prescribed prednisolone course, AECOPD hospitalization) or comorbid flare-ups (e.g., acute heart failure hospitalization), as registered in hospital patient records, were imputed when these were not reported via the e-diary. The patterns included bullets representing any extent of symptom worsening, and bars covering the episodes fulfilling definitions of acute exacerbations of COPD (AECOPD) and flare-ups of CHF, anxiety, and depression [8,28]. Potential self-reported triggers for AECOPDs or comorbid flare-ups (Text box 1), were added manually on the starting days of either an AECOPDs or comorbid flare-up using symbols. For additional context of time, months were added. This format was chosen to facilitate understanding of symptom deterioration in an accessible and analogical way for lay participants, sometimes with limited health literacy, as these simpler visualizations were preferred and have been shown before to better facilitate comprehension compared with more technical alternatives such as time-series charts [30].

**Text box 1. Description of the RE-SAMPLE international prospective cohort study.**

The RE-SAMPLE study is an international prospective cohort study, with participants from hospitals in the Netherlands, Estonia, and Italy [27]. The main objective of the RE-SAMPLE study is to identify parameters from real-world data that can predict disease deterioration of COPD and comorbidities. In the Netherlands, RE-SAMPLE included patients aged 40 + years who were smoker or ex-smoker, having a diagnosis of COPD according to GOLD criteria (i.e., postbronchodilator FEV_1_/FVC < 0.7 [3]), having at least one comorbidity (i.e., diabetes, CHF, ischemic heart disease, paroxysmal atrial fibrillation, anxiety, depression, obstructive sleep apnea) or at least two risk factors for developing additional morbidity (i.e., active smoker, hypertension, hypercholesterolemia, BMI < 18.5, BMI > 30, dialysis), having home access to internet, and able to understand, read and write Dutch. Patients either receiving palliative care or maintenance antibiotic therapy, having another lung disease (e.g., asthma), suffering from severe psychiatric illness (e.g., schizophrenia), or with cognitive impairment (i.e., MMSE <24 [31]) were excluded. At the Dutch study site, 71 patients were included between 2022 and 2024 after signing informed consent. Patients were followed until drop-out or the end of 2024 with lung function measurements scheduled at baseline and each six months during the study. Furthermore, patients kept an e-diary in which they were asked every 24 hours whether symptoms had increased (i.e., yes or no) compared to their stable level of symptoms. If answered ‘yes’, the extent to which symptoms had increased (i.e., no, slight, or significant increase) were reported specifically per disease (for COPD: dyspnea, sputum purulence and color, fever, coughing, and wheezing; for CHF: nocturnal dyspnea, swelling of ankles or abdomen, sudden overnight weight increase; for anxiety: anxiousness; for depression: depressive feelings). Per disease, patients were also asked to report on perceived triggering factors for symptom deterioration via the e-diary. Patients’ vital signs (e.g., heart rate) were semi-continuously monitored with wearables.

**Abbreviations**: COPD: chronic obstructive pulmonary disease; GOLD: Global Initiative for Chronic Obstructive Lung Disease; FEV_1_: forced expiratory volume in 1 second; FVC: forced vital capacity; CHF: chronic heart failure; BMI: body mass index; MMSE: Mini-Mental State Examination.

### Data collection

The semi-structured interviews were conducted (BR, SvD) between November 2024 and January 2025 in the hospital (patients and HCPs), at home (patients) or online via Microsoft Teams (HCPs). For the patient interviews, a topic guide was constructed based on literature and used to discuss personalized (multimorbid) care and the perceived added value of disease pattern visualizations (see S1 File) in each interview: 1) disease symptoms and their impact [32,33], 2) relatedness of COPD with comorbidity [34], 3) patient-centered organization of disease management and healthcare [32,35,36], 4) decision-making process [37–39], 5) self-management strategies [32,40–42], 6) use of their individual patient pattern of COPD and comorbid symptom deterioration [32,43]. For HCPs, this topic guide (see S1 File) included: 1) management and treatment of diseases [32], 2) organization of multidisciplinary healthcare [36,44], 3) decision-making process [37–39], 4) self-management behavior of patients [32,40,41], 5) the use of patterns of COPD and comorbid symptom deterioration [43] – a selection of patterns was discussed.

In-between interviews, the patient and HCP topic guides were regularly assessed (BR, SvD) to determine whether target questions could be revised for more meaningful responses or if any needed to be added to or removed from the topic guide. After thorough evaluation, no major adjustments were made. Participants did not receive their interview transcript. Qualitative analysis of the interviews was started after collection of four patient and four HCP interviews. Data collection through the interviews was stopped when data saturation was reached [45].

### Data analysis

Given the explorative nature of this study, an inductive approach (i.e., researchers derive overarching themes from the data) was chosen for the thematic analysis of the interviews. Following such an inductive approach, the first coder (BR) searched for first-order concepts in the transcript quotes, which were checked by the second coder (SvD), in accordance with the Gioia methodology [46]. These first-order concepts were clustered into second-order themes by the first coder, which was also checked by the second coder. Finally, overarching themes (i.e., the third order) emerged from clustered second-order themes, and were discussed among coders [46]. Atlas.ti 24.2.1 was used for the thematic analysis of the interviews [47]. In addition, relevant patient characteristics (e.g., sex, smoking status, educational level) were extracted from the RE-SAMPLE study database to support interpretation of the qualitative analysis.

## Results

**Fig 1:**
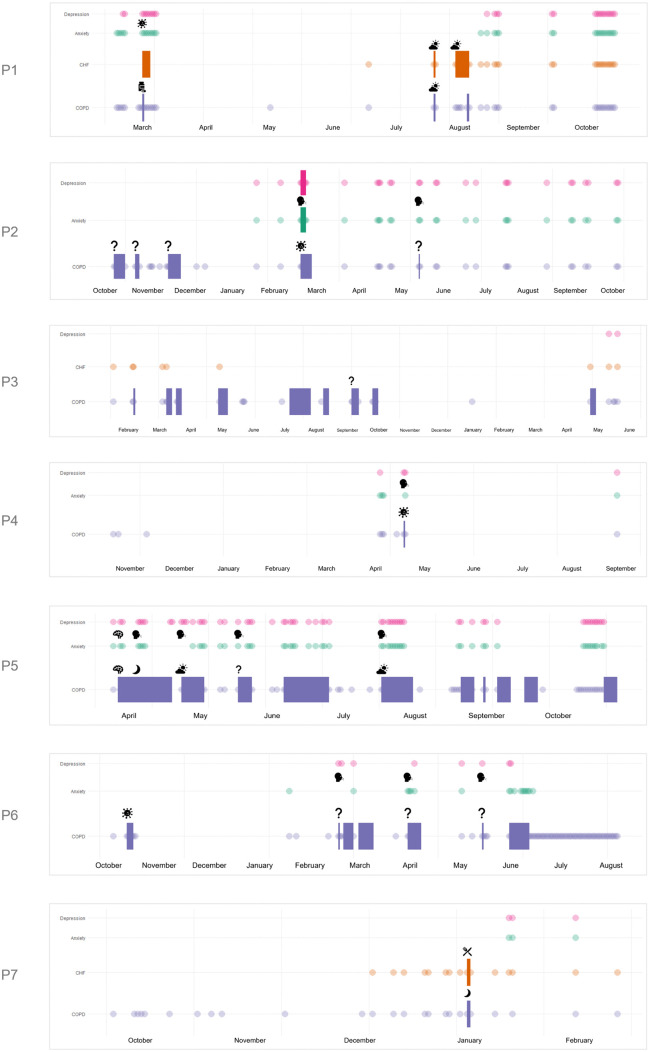
Patterns of symptom deterioration with corresponding reported perceived triggers, as discussed in the patient interviews. **Legend: ** acute exacerbation of COPD, acute heart failure episode, flare-up of anxious feelings, flare-up of depressive feelings, COPD symptom deterioration, CHF symptom deterioration, anxiety symptom deterioration, depressive symptom deterioration, illness, medication use, air quality and weather conditions, do not know, physical complaints, stress, sleep quality or fatigue, dietary changes. Note: total duration of follow-up (in days) differred between patients and ranged from 146 to 851 days. The patterns as discussed in the interview are shown in the right column, the pictograms represent the triggers for exacerbations/flare-ups as perceived by patients themselves.

In total, seven patients were included in this study (four men, age range 69–80 years) (Table 1). All suffered from moderate to very severe COPD (i.e., GOLD II-IV). Three patients suffered from comorbid CHF, for which they had reported deterioration of their symptoms during varying lengths of follow-up ranging between July 2022 and November 2024. All seven patients had reported on symptom deterioration of anxiety and/or depression. Patient characteristics are shown in Table 1. Their personal patterns of symptom deterioration with corresponding reported perceived triggers, as discussed in the patient interviews, are shown in Fig 1.

**Table 1:** Patient characteristics at the time patients were interviewed.

Participant ID	Sex	Age	COPD severity (GOLD category [3])	Smoking history (pack-years) & status	Chronic comorbidities	Educational level
**P1**	♀	71	2 – moderate	46 Ex smoker	CHF, IHD, DM2, OSA, HPN, ANX	Senior high school
**P2**	♂	77	3 – severe	75 Ex smoker	IHD, PAF, OSA	Senior high school
**P3**	♂	80	4 – very severe	13 Ex smoker	CHF, IHD, OSA, HPN, PVD	University
**P4**	♂	78	2 – moderate	71 Ex smoker	IHD	Senior high school
**P5**	♀	74	3 – severe	21 Current smoker	OSA	Junior high school
**P6**	♀	73	3 – severe	46 Ex smoker	DM2	Junior high school
**P7**	♂	69	2 – moderate	51 Ex smoker	CHF, DEP	Elementary school

**Abbreviations**: COPD: chronic obstructive pulmonary disease, GOLD: Global Initiative for Chronic Obstructive Lung Disease, P: patient, ♀: female, ♂: male, IHD: ischemic heart disease, DM2: diabetes mellitus type 2, OSA: obstructive sleep apnea, HPN: hypertension, PAF: paroxysmal atrial fibrillation, ANX: anxiety, CHF: chronic heart failure, HCL: hypercholesterolemia, PVD: peripheral vascular disease, DEP: depression.

Seven HCPs (one pulmonologist, one pulmonary nurse, one cardiologist, one cardiology resident, one cardiac nurse practitioner, and two medical psychologists) participated in this study. Six of them were female. The patterns P1 and P2 (Table 1) were discussed with the HCPs of the pulmonology and medical psychology and the patterns of P1 and P7 (Table 1) with HCPs from the cardiology department.

**Fig 2:**
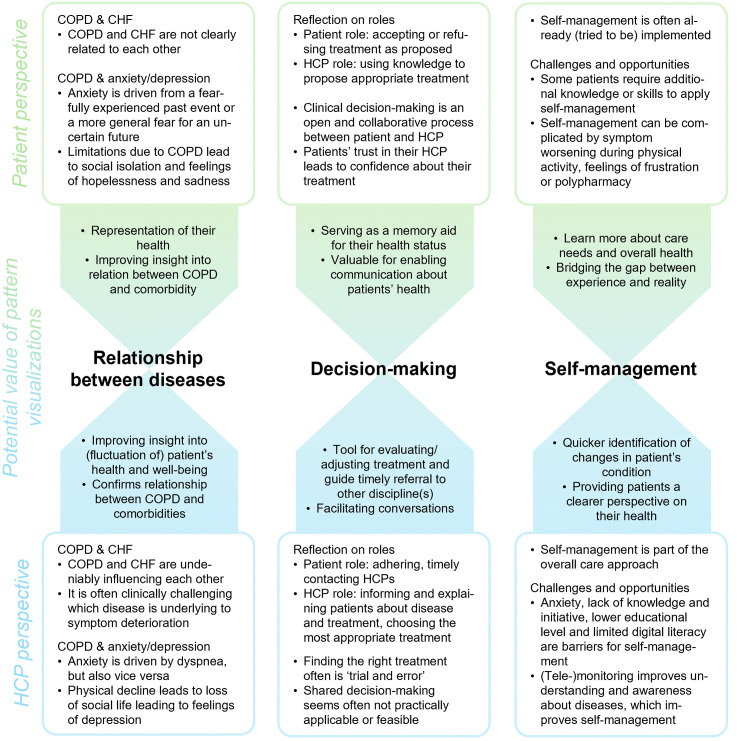
Summary of the results from the qualitative analysis described per identified theme: 1) relationship between diseases, 2) decision-making, and 3) self-management. For each theme, patient and HCP perspectives are described, bridging the two by the potential value of pattern visualizations. **Abbreviations:** COPD: chronic obstructive pulmonary disease; CHF: chronic heart failure; HCP: healthcare professional.

Through thematic analysis, three overarching third-order themes were identified in the patient and HCP interviews: 1) relationship between diseases, 2) decision-making, and 3) self-management. Each of the third-order themes is described from the patient and HCP perspective, respectively using quotes to support and illustrate the findings. Each theme section is ended describing the perceived value of visualizing symptom deterioration for supporting multimorbid disease management from both perspectives. A summary of the findings is presented in Fig 2, depicting the patient and HCP perspectives on each theme and what elements in pattern visualizations potentially bridges the differences between these two perspectives.

## Theme 1: Relationship between diseases

### Patient perspectives – COPD symptoms clearly influence mental health and well-being

The relationship between COPD and CHF was perceived differently. One patient saw the heart and lungs as directly connected, whereas this was not recognized by two other patients with CHF. The relationship between COPD deterioration and anxious feelings is clear, and can originate from fearful experiences during previous AECOPDs or a more general fear for an uncertain future disease course.

*“At that moment, I was sitting on a chair and unable to do anything. (…) Reflecting on it now, I remember the fear I had that I would experience the same thing [exacerbation] again. I was anxious about reliving that sensation, the intense tightness.” – P6*

Patients acknowledged that limitations due to COPD can lead to feelings of hopelessness and sadness. Also, they become more socially isolated because of their disease(s).

### HCP perspectives – relation between COPD and comorbid symptoms complicates care

All HCPs in pulmonology and cardiology recognized the connection between COPD and CHF. Three HCPs mentioned that COPD can trigger CHF deterioration or vice versa.

*“If you have a COPD exacerbation, the pressures in your circulatory system increase. That effects your heart, which might cause more fluid retention. This is a quite logical chain of events. Vice versa, if there’s fluid around the lungs, it can trigger bronchospasms, which can exacerbate COPD.” – HCP4*

The HCPs also touched upon the clinical challenge of differentiating between COPD and CHF.

*“COPD and CHF share common symptoms, such as shortness of breath or fatigue. Therefore, when a patient reports these symptoms, it can be quite challenging to determine which condition is causing these symptoms. – HCP3*

All HCPs recognized a clear link between COPD and/or CHF and mental health. According to them, physical symptoms (mainly dyspnea) can trigger anxious feelings. Anxiety was assumed to originate, besides from symptoms only, also from previous experiences.

*“When shortness of breath occurs, people often say: “I have been admitted before and that experience truly scared me”. You notice that this plays a role when they get similar complaints the next time, the fear: “Oh no, will it be like that experience again, and shall I have to be admitted again?” – HCP2*

Also vice versa, anxiety was mentioned to provoke dyspnea. HCPs acknowledged that reduced mobility and energy limit, related to COPD and/or CHF, can affects patients’ social network and lead to feelings of sadness or depression.

### Using visualizations – improving patient and HCP insights into (concurrent) presentation of COPD and comorbid symptoms

Patients felt that visualizations represent their health status and they could serve as a memory aid. For them, it provided additional insight into their disease and how it can change over time. For the majority of patients, the visualization strengthened their understanding of the relationship between COPD and comorbidities.

The visualizations did not surprise HCPs, since they recognized the simultaneous occurrence of different diseases’ symptoms from practice, as well as the triggers patients reported.

*“It [visualization] confirms what you encounter in daily practice, showing that there is indeed a connection between these conditions.” – HCP2*

HCPs reported that the patterns could enhance the patients’ understanding of their diseases, but also that they can help HCPs in identifying patients’ health status from the perception of the patient.

*“I really find this [visualization] very relevant. It’s interesting, you see… It’s clear that the medical perspective is different from the patient perspective. That’s a fact. So the more I understand the patient perspective, the better I can think along with them. I mean, I know the medical part, the technical side — but if this is also going on, then I can offer things like, “I can refer you,” or “we can try this.” You can respond in a much more targeted way.” – HCP3*

## Theme 2: Decision-making

### Patient perspectives – based on trust on HCP passively contributing to clinical decisions

Decision-making was generally seen as a process in which the HCP proposes a treatment (plan), and the patient either accepts or refuses. They consider the HCP responsible for proposing appropriate treatment options.

*“They [the HCPs] are the experts, so I trust their judgment and rely on their expertise.” – P3*

Patients did, overall, not consider an active role for themselves in clinical decision-making, though they feel the room to raise concerns (e.g., for side-effects).

*“If it [a proposed treatment] really goes against my will, then I will say something about it. (...) But they [the HCPs] know what they are doing, I assume.” – P1*

All patients saw decision-making as a communicative and collaborative process with the HCP and expressed satisfaction.

*“What is most important to me is, what I just said, that I have a doctor who is approachable, who listens to you and who also gives you an answer to that. And then you come to a treatment plan together, that is just (…) a collaboration, I think, and then you go home with a good feeling.” – P4*

### HCP perspectives – challenging to fully involve patients in decisions, multidisciplinary collaboration is important yet limited

From the HCP perspective, treatment decisions are based on their understanding of the patient’s needs. Also factors including age, comorbidities, severity of symptoms, disease progression, and side-effects are taken into account. Furthermore, motivation, treatment adherence, lifestyle, and inhalation capacity are considered. Multiple HCPs reported that finding the most appropriate treatment sometimes is trial-and-error.

*“When a patient experiences multiple side-effects from various medications (…) it becomes a continuous process of evaluating and identifying treatments that are both effective and well-tolerated.” – HCP2*

Medical psychologists considered decision-making patient-centered, with continuous collaboration between the patient and HCPs aiming on making patients active participants of their care, which was seen essential for treatment success. The HCPs working in the pulmonology and cardiology departments reported that, in many cases, it is challenging to fully involve patients in decisions made due to limited treatment options.

*“The idea of shared decision-making is somewhat artificial, because that’s not how reality works. (…) For many conditions, you don’t have a choice between treatment options one, two, and three. There simply is a treatment, and you can either choose to take it or not.” – HCP3*

In cases where they consider it more feasible, shared decision-making is applied.

*“An area where decisions are made more collaboratively is the initiation of oxygen therapy, as this is a treatment patients need to fully understand and consent to. (…) This decision-making process is more strongly aligned with the principles of shared decision-making.” – HCP1*

HCPs shared that they feel that, currently, most patients rely on HCP expertise for making decisions. The extent to which patients engage in decision-making varies and seems more for patients with a higher socio-economic and/or educational background. HCPs felt that patients themselves also have to take responsibility with regard to disease management by following medical advice, timely care-seeking, and asking questions.

Comorbidities do not necessarily change the care provided from a certain discipline. Hospital care was reported to be fragmented mostly, which was not considered efficient per se.

*“Healthcare is currently structured in such a way that it consists of separate “silos”, which I sometimes see as a problem. (…) It makes healthcare inefficient.” – HCP5*

Currently, multidisciplinary collaboration in the hospital seems to primarily consist of telephone calls, referrals and delegation of tasks to other specialties.

*“The tricky thing about our electronic patient record system is that you can easily send orders to each other, and people like doing that because: once they’ve sent their order, it’s on someone else’s desk. So, my impression is that it is a good system – one has insight into everything – but it also makes it really easy to just quickly pass orders.” – HCP6*

HCPs expected that more intensive multidisciplinary collaboration initially requires additional time, but ultimately leads to time savings and better patient outcomes.

*“I believe that collaboration and thorough consultation are very important and ultimately cost-saving. Of course, it takes time (…), but in the end, it is effective.” – HCP7*

HCPs feel that their high workload, as well as lack of institutional encouragement, hinders advances in multidisciplinary care.

### Using visualizations – facilitating patient-HCP conversations and multidisciplinary collaboration from a shared perspective

Patients reported that the visualizations could provide opportunity for dialogue with their HCP, functioning as a conversation starter.

*“When you see it on paper, then it becomes real.”*

*– “And then you talk about it more easily.” (Partner)*

*“Yes, maybe so.” – P6*

Visualizations can perhaps bridge the gap between the perceived health by giving a more realistic overview with data collected real-time.

*“To me, it [symptoms] just quietly continues. And yes, I don’t feel well, but that’s just part of me. And when I see this, I think: ‘yes, that makes sense’”*

*– “Yes, and do you think this could help a healthcare provider better understand how you are doing?” (Interviewer)*

*“Yes, I think so. I don’t really reflect on it myself. It happens, and then it’s over, and I forget about it.” – P1*

One patient expressed that it provided the ability to explore their diseases together with an HCP.

*“It [visualization] gives us something to chat about, like: ‘How do you feel about this? I see this, and I see that. Can you explain that? How does that work for you?’” – P7*

HCPs reported that visualizations could facilitate meaningful discussions between them and a patient, too. The fact that visualizations represent daily reality makes it useful for periodical evaluation and perhaps for adjusting ongoing treatments.

“*Often, when I ask a patient ‘How did you do last week?’, they don’t always remember exactly how it [acute heart failure episode] started, on which day, or how severe their symptoms were. (…) Maybe we could adjust the treatment a bit more based on that.” – HCP4*

HCPs also highlighted the possibility of visualization of symptom deterioration over time to educate patients about their disease and care. It helps identifying patient needs outside of an HCP’s own discipline potentially improving engagement of HCPs from other disciplines and contribute to timely referrals.

*“This [visualization] allows you to provide patients with more specific information, moving beyond general explanations, and enables you to inform them more effectively and concisely about the care they require. It could also facilitate earlier referrals, ensuring they [patients] are directed to appropriate services.” – HCP5*

Although they saw value of using symptom deterioration patterns for decision-making, they reported that lack of detail in visualizations could limit effective use. Also the limited time for consultation was reported as a barrier for implementation.

## Theme 3: Self-management

### Patient perspectives – self-management gives autonomy, but is challenging in a multimorbid context

Current self-management strategies, as reported by patients, included to stay physically active, maintain a healthy diet, quit smoking, and pace their energy. Being able to respond to symptom deterioration themselves, for example by taking additional medication or doing breathing exercises, gives patients a sense of autonomy with regard to their care.

*“I have the same dilemma when it comes to taking prednisolone. I don’t take it easily. I am currently constantly struggling with the question: should I take it or not yet? (…) Ultimately, it’s my decision. The very first time, the doctors decided for me, the first time I took it. Until I was told: ‘You should always make sure you have it at home.’ So now, I always keep a box in the house.” – P6*

Most patients felt confident in self-managing their diseases having sufficient knowledge about their disease and self-management skills. Barriers for effective self-management were also reported by patients, such as increased symptoms during physical activity challenging an active lifestyle.

*“I am thinking about it because I know that the way I am living right now is not good. I do not get enough exercise; I use my mobility scooter for everything. But I also cannot do without. I get so out of breath. Even if I have to walk from here to the garbage bin, I’ll still take the scooter.” – P1*

Also, struggles with maintaining a healthy weight were expressed. In response to symptom deterioration, some patients reported struggles to perform breathing exercises or feelings of frustration when ‘nothing seems to help’.

*“Wednesday, well, I had done some exercise in the morning, and we had plans to go out in the evening. So I thought, let’s take it easy the afternoon since we’re going out later. And around 6 o’clock it [severe breathlessness] started. So I was taking it slow, sitting down… And then in the evening, I was so short of breath still. It just didn’t work. I was really breathless. I simply couldn’t go out. And then I get so frustrated. That it just doesn’t work.” – P6*

Additionally, complexity of multiple medications was reported to lead to doubts about their effectiveness and to confusion.

*“I had been very sick, (…) which caused my potassium levels to drop dangerously low. I should’ve stopped that pill, and I didn’t. But I have no idea which pill exactly I should stop taking. I wouldn’t know.” – P1*

### HCP perspectives – understanding of one’s diseases is essential for successful self-management

Self-management was seen as an important component of care in patients with multimorbid COPD, according to all HCPs. HCPs provide information and advice about self-management strategies with regard to an active lifestyle, a nutritious diet and quitting smoking. Additionally, HCPs in pulmonology offer practical guidance regarding breathing exercises and inhalation technique. All three disciplines are increasingly implementing disease and symptom monitoring through mobile apps to increasingly involving patients in managing their own disease. Apps were also expected to lead to better understanding of their diseases in patients, which was mentioned to be essential for self-treatment.

*“Being able to take additional diuretics [in response to increased CHF symptoms] independently is only possible if patients have a good understanding of their disease.” – HCP 3*

According to HCPs, self-management facilitates stronger patient autonomy but they keep on seeking ways for optimal self-management support. Several HCPs mentioned that advice often needs to be repeated without observable effects and that educational brochures or personalized self-management action plans are often not being used.

*“Almost all of them [patients with COPD] have an action plan. But can they actually recall it? Very rarely. (…) It’s really a challenge, because they just put it in a drawer.” – HCP1*

Not all patients seem to be fit for self-management according to HCPs. Patients have to be interested and motivated but also disciplined. Also, lack of initiative, lower education level, and limited digital literacy – in case of telemonitoring – can complicate self-management. Furthermore, HCPs reported that the complexity of multimorbidity also hinders successful self-management.

*“When it comes to self-monitoring and understanding the disease, it’s really about the whole disease process. Ideally, though, that also requires a certain level of intelligence. With heart failure, for example, patients often have at least four medications, and a list of ten isn’t uncommon. (…) If you really know why you’re taking your medications and for which diseases, you’re more likely to take them correctly and consistently. So, it’s all connected, in a way.” – HCP3*

### Using visualizations – offering patients a clearer perspective on their health and care needs

The majority of patients did not specifically touch upon the use of the visualization for self-management. One patient shared that visualizations could help in changing and/or maintaining self-management behavior. Since such visualizations could provide a better idea of the impact of certain choices on their health, it could lead to more proactive disease management by patients themselves.

*“With the COPD, I’m glad I quit smoking, even though it’s still very challenging. And yes, I know I need to speak up if things aren’t going well because I just need time to adjust. (…) This [visualization] really shows me that I’m sicker than I had previously admitted to myself.” – P7*

HCPs mainly reported that, for patients, the visualizations could lead to a clearer understanding of their disease. This might improve self-management when patients are motivated to take more ownership regarding their health.

*“I think it would mainly have a positive impact on self-management. That would be the biggest advantage in my opinion: I think it would help patients gain a clear understanding of what is going on at the moment. On which days do they really experience symptoms? And what exactly are the triggers?” – HCP4*

HCPs also expressed doubts regarding the burden of daily symptom tracking on the patient. Additionally, patients might be discouraged when behavior changes are not reflected by improvements in the visualizations.

*“A patient may feel like a failure if they are unable to manage their condition effectively, which is not the intended outcome. For instance, patients might think: ‘I have this issue, I struggle with anxiety, and I still can’t get it under control. How frustrating! What a failure I am.’” – HCP7*

## Discussion

This study aimed to explore how patterns of COPD and comorbid symptom deterioration via remote monitoring could support personalized multimorbid COPD management as perceived by patients and HCPs across various medical disciplines. Three elements of personalized multimorbid COPD management were identified through qualitative analysis: 1) relationship between diseases, 2) decision-making, and 3) self-management, all of which can potentially be supported by pattern visualizations. This study shows that insight into patterns of symptom deterioration via remote monitoring might enhance patients’ understanding of their diseases and the relationship with each other. Additionally, such insights might improve shared decision-making between patients and their HCPs, and aid self-management strategies. The patient and HCP perspectives on multimorbid COPD management were not always well aligned, underscoring the need to bring the two together. The results of this study showed that pattern-based monitoring has the potential to enable more collaborative, personalized care and improve multimorbid COPD (self-)management, thereby, bridging the gap.

Visualization of multimorbid symptom patterns could enhance patients’ understanding of the relationship between COPD and comorbidities. This was considered crucial by the HCPs to improve care in these complex patients, for example, by allowing self-management in response to symptoms. This finding seems generally supported by earlier research, indicating that more knowledge in patients improves their health-related outcomes and use of healthcare resources [48]. However, it should be noted that increased patient knowledge will not automatically lead to more appropriate decisions regarding treatments or disease management [49]. The transfer of knowledge to adequate disease management also requires other factors that contribute to patient empowerment [50]. This idea is supported by a systematic review: not only patient knowledge is necessary for equal participation of patients in clinical decision-making, but also power [51]. Patient empowerment seems particularly difficult to address with educational means since its dependence on the context of the patient, HCP, and the system in which they operate [51]. Therefore, visualizations of symptom deterioration patterns should not be seen as the holy grail, but can merely be part of the solution for patients with multimorbid COPD.

Our study highlights that, while patients perceive decision-making as a shared process, they tend to take a passive role, which aligns with previous research [52]. In our study, HCPs also noted patients’ passivity in decision-making. Literature reports that it can be difficult for HCPs to adequately assess patients’ care needs when they are not actively engaged in decision-making [12,53]. Although HCPs in our study generally aimed for collaboration with patients, practical constraints can hinder shared decision-making. Importantly, our findings suggest that patients perceive pattern visualizations valuable for facilitating discussions by aiding them in expressing and remembering their experiences. Also in other studies such visualizations have been shown to improve understanding of patients’ care needs [54–56]. Furthermore, they showed that representative day-to-day health data visualizations aids HCPs in assessing a patient’s actual health status and specific needs [54–56]. Visualizations of multimorbid COPD patterns can, thus, perhaps bridge the decision-making gap between patients and HCPs. Additionally, HCPs in our study reported limited joint decision-making with multiple clinical disciplines although they recognized its potential to enhance patient care, which aligns with previous research [57]. HCPs saw visualizations as a potential starting point for engaging other specialties, particularly for timely referrals in case of concerns about patients’ mental health. However, time constraints were reported as key barrier to effective multidisciplinary collaboration.

Patients attempt to engage in self-management primarily through lifestyle improvements. To illustrate, one patient reported to be motivated by the pattern visualization to persist in quitting smoking. Health data visualization was also considered valuable to evaluate whether such behavioral changes lead to the desired effect in patients [55]. Our study identified limited patient action in response to worsening symptoms with taking additional medication themselves and HCPs noted that patients regularly forget about self-treatment action plans. Although early treatment has shown to shorten AECOPD duration and reduce the risk of emergency department visits and hospitalizations [16,58], according to HCPs, the patients seem to miss this opportunity and ‘wait and see’. By helping patients with earlier recognition of their care needs and actively engaging and empowering them in their disease management [55,56], real-time visualizations may foster timely self-treatment initiation, ultimately improving health outcomes. Also, visualizing patient-reported triggers of symptom deterioration or life events has been shown to improve patients’ understanding of their disease in patients with hypertension before [56]. Therefore, implementing perceived triggers for disease fluctuation into the pattern visualizations could serve a comparable purpose in multimorbid COPD.

An important strength of our study is the mixed-methods design. This approach allowed for integrating quantitative symptom monitoring data into the qualitative interviews. Patients were presented with a personalized visualization of their multimorbid COPD patterns, created using self-reported symptom deterioration data. This approach enabled reflective discussions by supporting to place abstract experiences (e.g., increased breathlessness) in their personal context (e.g., crowded birthday party in combination with hot weather) in order to reach a better understanding of their disease. Moreover, the pattern visualizations were used to facilitate patient-centered discussions with HCPs, allowing for talking about individualized disease trajectories over time as reported by real patients. This enriched the data quality, but also exemplifies how qualitative data collection can be enhanced through the use of real-world digital health data. Another strength is the inclusion of both patient and HCP perspectives, spanning multiple medical disciplines. Analyzing both perspectives was considered valuable, since it is known that patient and HCP perspectives do not necessarily match [59]. By explicitly exploring both sides, this study was able to highlight gaps in understanding and communication that were otherwise unidentified. Finally, the study was continued until data saturation was reached, supporting robustness and sufficient depth of the findings.

Our study also has limitations. First and foremost, the typical multimorbid COPD management team consists of many more people than the patient and in-hospital HCPs only. Furthermore, the patients included were between 69 and 80 years of age and exclusively Dutch. While qualitative research does not aim to achieve statistical representativeness, the demographic scope of our study’s participants might limit transferability of our findings to patient groups from different age, cultural, and/or healthcare contexts. Similarly, the focus on hospital-based HCPs means that perspectives from community-based professionals such as general practitioners, physiotherapists, and informal caregivers, who play a key role in multimorbidity management, were not included. These limitations imply that the experiences and needs described in our study may primarily apply to older, hospital-treated patients, and caution is warranted when extrapolating to other populations or care settings. Second, the design chosen for the pattern visualizations may have influenced the results. Although simple visualizations valuably stimulated discussion during the interviews because of their accessibility, alternative visualization techniques (e.g., line or area charts) could possibly have directed different or additional insights. Although patients in another study seem to prefer simple and accessible visualization techniques [30], HCPs in our study reported that lack of detail in the visualizations might hinder applicability in practice. As a result, the findings primarily reflect participants’ interpretations of disease flare-up sequences and their time- and trigger-related contexts, while more detailed symptom fluctuations could have been of added value but were ignored. Third, as in all qualitative research, personal influence of the involved researcher on the interview data collected and the reporting is present. To provide maximal transparency about data collectors and the way interviews and analyses were conducted, the COREQ checklist [26] was used. Furthermore, the degree of subjectivity was minimized by involving multiple researchers, who operated independently, in the qualitative analysis. Finally, depth of discussions varied across interviews due to time constraints and differences in how extensively participants shared their experiences. A different data collection method, such as focus groups with multiple patients or HCPs, might have led to additional insights.

Future research could further explore how visualization of patterns in health data should be optimized for clinical decision-making. Additional insights can be found via other data collection methods, such as surveys or focus groups, or by iterative longitudinal qualitative designs. The latter may yield insights into how visualizations are interpreted and used over time, or how patient behavior changes or adjustments in healthcare can be made. The inclusion of a broader group of healthcare providers (e.g., general practitioners, informal caregivers) would add to a comprehensive understanding of how multimorbid COPD management can be supported with health data visualization across care settings. Patients and HCPs in our study shared their opinions on the potential value of integrating pattern visualizations in healthcare, but whether they can actually change behavior and have positive effects on health and wellbeing is still unknown and should be investigated [55]. Given that real-world data collection is increasingly becoming part of routine care but often focuses on separate diseases, future research should investigate how multimorbid visualizations can support more holistic approached in chronic care. This may facilitate a shift from disease-specific management to more person-centered care, enabling earlier recognition of symptom deterioration and improving shared decision-making and self-management. However, successful implementation requires attention for design and usability, alignment with patient and HCP needs, and how these can be embedded into existing clinical workflows as a valuable addition.

## Conclusion

This study highlights the potential of pattern visualizations of symptom deterioration through remote monitoring for supporting personalized multimorbid COPD management. By providing insight into the relationship between COPD and comorbidities, these visualizations can improve patient understanding, facilitate shared decision-making between patients and HCPs, improve in-hospital multidisciplinary collaboration, and aid self-management strategies. Because patient and HCP perspectives do not always align, pattern-based monitoring can promote more collaborative and person-centered care. Our study showed that visualizations of multimorbid disease patterns allow for clearer communication between patients and HCPs, and improves understanding of a patient’s care needs. Future research should further explore the impact of monitoring data-based pattern visualizations on behavior, health outcomes, and the integration of such visualizations into existing workflows.

## Supporting information

S1 File Supplementary-Material. (PDF)
