# Supplementary material for: Patterns of symptom deterioration can support multimorbidity management in COPD: Perspectives of patients and healthcare professionals
Source: PLoS One. 2025 Dec 17;20(12):e0338888. doi: 10.1371/journal.pone.0338888 (PMC12711060; doi:10.1371/journal.pone.0338888)
Supplement: S1 File — (PDF) [file pone.0338888.s001.pdf]

Supplementary materials

Supplementary material 1 – COREQ checklist

The Consolidated Criteria for Reporting Qualitative Studies (COREQ): 32-item checklist

**Domain 1: research team and reflexivity**		
**Personal characteristics**		
1. Interviewer/facilitator	Which author(s) conducted the interview or focus group?	BR conducted the interviews. SvD supervised the interviews with the patients.
2. Credentials	What were the researcher's credentials? (e.g. PhD, MD)	BR: BSc at the time the interviews were conducted<br>SvD: MSc BA at the time the interviews were conducted
3. Occupation	What was their occupation at the time of the study?	BR: Master student Health Sciences, University of Twente<br>SvD: PhD candidate and epidemiologist, University of Twente
4. Gender	Was the researcher male or female?	Both BR and SvD were female.
5. Experience and training	What experience or training did the researcher have?	BR and SvD conducted qualitative research for both their BSc theses. Qualitative research was part of BR's and SvD's bachelor programs. In preparation for this project, BR and SvD followed a short e-course on qualitative research, which was organized by the hospital where the research was carried out.
**Relationship with participants**		
6. Relationship established	Was a relationship established prior to study commencement?	Prior to the recruitment, researchers had no professional or personal relationship with the participating patients.<br>BR had no profession or personal relationship with the HCPs prior to recruitment
7. Participant knowledge of the interviewer	What did the participants know about the researcher? (e.g. personal goals, reasons for doing the research)	All participants signed informed consent before participation in the interview. As part of this informed consent, all participants were informed about the research goal. No information was shared about the personal goals of executing this study (e.g., part of thesis completion). Their was no care-related relationship between the patients and the interviewer (BR or SvD). Their was no professional relationship between the HCPs and the interviewer (BR).
8. Interviewer characteristics	What characteristics were reported about the interviewer/facilitator? (e.g. bias, assumptions, reasons and interests in the research topic)	With the patient interviewees, it was shared that BR and SvD were researchers within the RE-SAMPLE project. BR was a master student Health Sciences at the time the interviews were conducted. This was shared with the HCP interviewees. BR did not have a medical background, which was beneficial because of her ability to ask 'naïve' questions. This way, she could ask for clarification where needed, which resulted in rich data with minimized bias. BR, of course, did preparatory work on the topic.
**Domain 2: study design**		

**Theoretical framework**		
9. Methodological orientation and theory	What methodological orientation was stated to underpin the study? (e.g. grounded theory, discourse analysis, ethnography, phenomenology, content analysis)	The study was conducted without a predefined theoretical framework. The topic guide designed for this study was based on literature (see supplementary material for references).
**Participant selection**		
10. Sampling	How were participants selected? (e.g. purposive, convenience, consecutive, snowball)	We used purposive sampling based on criteria mentioned in the method section of the article.
11. Method of approach	How were participants approached? (e.g. face to face, telephone, mail, e-mail)	Patients were approached face-to-face during meetings organized for the RE-SAMPLE study or via telephone. HCPs were approached face-to-face or via e-mail.
12. Sample size	How many participants were in the study?	7 patients and 7 HCPs.
13. Non-participation	How many people refused to participate or dropped out? Reasons?	No patients who were approached refused to participate. One HCP who was approached refused to participate.
**Setting**		
14. Setting of data collection	Where was the data collected? (e.g. home, clinic, workplace)	The patient data was collected in the hospital or at the patient's home when requested. The HCP data was collected in the hospital or via Microsoft Teams when requested.
15. Presence of non-participants	Was anyone else present besides the participants and researchers?	Two patients were accompanied by their spouse. One patient was accompanied by the daughter. During other interviews, there were no people present during the data collection besides participants and researchers (BR & SvD).
16. Description of sample	What are the important characteristics of the sample? (e.g. demographic data, date)	Relevant demographics of the patients have been reported in Table 1.
**Data collection**		
17. Interview guide	Were questions, prompts, guides provided by the authors? Was it pilot tested?	The topic lists used in the interview are provided (see supplementary material). The topics were accompanied with starting questions and potential in-depth questions.
18. Repeat interviews	Were repeat interviews carried out? If yes, how many?	There were no repeat interviews conducted with the same participants.
19. Audio/visual recording	Did the research use audio or visual recording to collect the data?	All interviews were audio recorded using Microsoft Teams with permission (as signed for by means of informed consent) of the participants.
20. Field notes	Were field notes made during and/or after the interview or focus group?	SvD made field notes during the interviews with patients. BR made field notes during the interviews with HCPs.
21. Duration	What was the duration of the interviews or focus group?	The average duration of the interviews with patients ranged from 27 to 57 minutes, with an average duration of 42 minutes. The average duration of the interviews with HCPs ranged from 21 to 51 minutes, with an average duration of 37 minutes.
22. Data saturation	Was data saturation discussed?	Yes. After data collection of the first 4 patient and HCP interviews, after each additional interview, the findings were reviewed. Data collection through new interviews was ended when BR and

		SvD were convinced new interviews would not contributed to new findings (i.e., when data saturation was reached). This point was reached after 7 patient and 7 HCP interviews.
23. Transcripts returned	Were transcripts returned to participants for comment and/or correction?	Transcripts were not returned to participants, except when particularly requested. No corrections were made by the study's participants.
**Domain 3: analysis and findings**		
**Data analysis**		
24. Number of data coders	How many data coders coded the data?	Two researchers (BR, SvD) coded the data. Disagreements were discussed until consensus was reached (BR, SvD).
25. Description of the coding tree	Did authors provide a description of the coding tree?	The coding tree was not provided as part of this publication, but can be retrieved upon reasonable request from the corresponding author.
26. Derivation of themes	Were themes identified in advance or derived from the data?	Themes were derived from the data using an inductive approach (i.e., from the data).
27. Software	What software, if applicable, was used to manage the data?	Microsoft Teams was used for initial data transcription. BR used the audio recording to manually finalize the interview transcripts. BR and SvD used Atlas TI for the coding of the interviews.
28. Participant checking	Did participants provide feedback on the findings?	No.
**Reporting**		
29. Quotations presented	Were participant quotations presented to illustrate the themes/findings? Was each quotation identified? (e.g. participant number)	Quotations were used to support and illustrate the findings, identified by a P or HCP with a unique number.
30. Data and findings consistent	Was there consistency between the data presented and the findings?	Yes.
31. Clarity of major themes	Were major themes clearly presented in the findings?	Each major theme (i.e., third order overarching theme) is described separately in the results section.
32. Clarity of minor themes	Is there a description of diverse cases or discussion of minor themes?	Within each major theme presented in the results section, minor themes (i.e., second order themes) are used to describe the major theme, supported with quotations.

BR: Bente Rodenburg, SvD: Sanne van Dijk, MSc: Master of Science degree, BSc: Bachelor of Science degree, BA: Bachelor of Arts degree, PhD: Doctor of Philosophy degree

Supplementary material 2 – Interview topic guides

Topic guide patient interview

1) symptoms of diseases and their impact (29,30)
   - Type of symptoms/complaints (29)
   - Triggering factors for increases in symptoms/complaints (29)
   - How symptoms affect daily life and functioning (29,30)

2) relatedness of COPD with comorbidity
   - Coherence between diseases (COPD – CHF, COPD – mental health, CHF – mental health)
   - Differentiating between diseases
   - Mental and physical health (31)

3) patient-centered organization of disease management and healthcare
   - Current management/treatment of diseases (29)
     - Opinion regarding current management/treatment of diseases
   - Discussing of complaints with HCP (32)
   - Multidisciplinary care (33)

4) decision-making process
   - Current decision-making process
     - Role/responsibility of HCP (34)
     - Role/responsibility of patient (34,35)
   - Shared decision-making (36)
   - Opinion regarding current decision-making process

5) self-management strategies (29)
   - Insight into own health and disease (29)
   - Dealing with symptoms/complaints (29)
   - Self-management (29)
     - Use of additional tools/technology (37)
     - Necessary skills and knowledge (38)
     - Challenges in dealing with symptoms/complaints (38)
   - Role/responsibility caregivers (29)
   - Role/responsibility social environment (e.g., partner, family, neighbors) (39)

6) the use of their own pattern of COPD and comorbid symptom deterioration
   - (Fluctuation of) symptoms/complaints over time (29)
   - Coherence between diseases
   - Recognition of pattern
   - Value of pattern (40)
     - Use/implementation of pattern by patient and HCP
     - Advantages of implementing pattern (40)

Topic guide HCP interview

1) management and treatment of diseases
   - Current management/treatment of diseases (29)
     - Opinion regarding current management/treatment of diseases (29)

2) organization of multidisciplinary healthcare (33)
   - Addressing comorbidities in disease management
   - Collaboration between medical disciplines (33)
     - The extent to which collaboration takes place (33)
     - Barriers for collaboration (41)
     - Ways in which collaboration is/should be supported (41)
   - Prerequisites for collaboration

3) decision-making process
- Current decision-making process
  - Role/responsibility of HCP (36)
  - Role/responsibility of patient (34,35)
- Interaction between HCP and patient (34)
- Personalizing treatment decisions
- Shared decision-making (36)
  - Barriers for shared decision-making (36)
  - Ways in which shared decision-making is/should be supported (36)
- Factors influencing decision-making (36)
- Opinion regarding current decision-making process

4) self-management behavior of patients (29)
- Insight into own health and disease (29)
- Self-management (29)
  - Use of additional tools/technology (37)
  - Necessary skills and knowledge (38)
  - Challenges in dealing with symptoms/complaints (38)
- Role/responsibility caregivers (29)

5) the use of patterns of COPD and comorbid symptom deterioration
- Coherence between diseases
- Recognition of pattern
- Value of pattern (40)
  - Use/implementation of pattern by patient and HCP
  - Advantages of implementing pattern (40)
  - Potential for multidisciplinary, personalized care and self-management (40)
